# Optimizing the Impact of Public-Academic Partnerships in Fostering Policymakers’ Use of Research Evidence: Proposal to Test a Conceptual Framework

**DOI:** 10.2196/14382

**Published:** 2019-05-24

**Authors:** Christina D Kang-Yi

**Affiliations:** 1 Center for Mental Health Perelman School of Medicine University of Pennsylvania Philadelphia, PA United States; 2 Leonard Davis Institute of Health Economics University of Pennsylvania Philadelphia, PA United States

**Keywords:** public-academic partnership, use of research evidence, youth, mental health, well-being

## Abstract

**Background:**

Previous research has reported that public-academic partnerships (PAPs) can effectively promote PAP leaders’ use of research evidence in improving youth outcomes. However, the existing literature has not yet clarified whether and how PAP leaders’ use of research evidence evolves along the PAP life cycle and whether PAP partners’ concordant perceptions of usefulness of their PAP has an impact on PAP leaders’ use of research evidence. Developing a conceptual framework that recognizes the PAP life cycle and empirically identifying contexts and mechanisms of PAPs that promote PAP leaders’ use of research evidence from the PAP life cycle perspective are imperative to guide researchers and policymakers to successfully lead PAPs and foster policymakers’ use of research evidence for improving youth outcomes.

**Objective:**

Utilizing an integrated framework of organizational life cycle perspective, a social partnership perspective, and a realist evaluation, this study examines the extent to which PAP development and PAP leaders’ use of research evidence can be characterized into life cycle stages and identifies PAP contexts and mechanisms that explain the progress of PAPs and PAP leaders’ use of research evidence through life cycle stages.

**Methods:**

Recruiting PAPs across the United States that aim to improve mental health and promote well-being of youth aged 12-25 years, the study conducts a document analysis and an online survey of PAPs to inform policymakers and academic researchers on the contexts and mechanisms to increase PAP sustainability and promote policymakers’ use of research evidence in improving youth outcomes.

**Results:**

Fifty-three PAPs that meet the recruitment criteria have been identified, and document review of PAPs and participant recruitment for the online survey of PAP experience have been conducted.

**Conclusions:**

This paper will help policymakers and researchers gain a deeper knowledge of the contexts and mechanisms for each PAP life cycle stage in order to optimize PAP leaders’ use of research evidence in achieving positive youth outcomes.

**International Registered Report Identifier (IRRID):**

DERR1-10.2196/14382

## Introduction

### Background

This proposed project aims to develop a conceptual framework to understand dynamic and complex public-academic partnerships (PAPs) and reveal contexts and mechanisms for each PAP life cycle stage in order to optimize PAP leaders’ use of research evidence in improving youth mental health and well-being. The proposed project defines a PAP as a partnership between the state and county policymakers (administrators and program directors) and researchers at academic institutes, formed to promote evidence-informed policymaking and practice. PAP leaders are policymakers who influence public identification of problems, design and implement programs, and make policy decisions as administrators and program directors.

This project focuses specifically on PAPs that aim to improve mental health and promote well-being of youth aged 12-25 years. Many psychiatric disorders such as mood disorders, substance abuse problems, and schizophrenia develop during adolescence [1]. About 20% of US youths aged 13-16 years will experience a psychiatric disorder during their lifetime [2-4], and the rates are higher among youth served by the public care sector [5-7]. PAPs are vital to improve the health and well-being of vulnerable populations [8-11]. PAPs seek to bridge the historic divide and disconnect that has evolved among researchers and policymakers and improve the degree to which the knowledge generated by researchers is utilized for the benefit of the individuals being served by the public care sector [12,13]. A critical means through which PAPs accomplish this aim is the use of research evidence. Research evidence is defined as relevant conceptual frameworks or reviews and empirical findings from systematic qualitative, quantitative, or mixed research methods projects [14]. Use of research evidence is defined as acquiring, evaluating, and directly applying research evidence [15,16] and conceptually using research [17,18] to understand the nature of and frame social and community problems; to design and implement public services and programs; and to make policy decisions [19,20].

Previous research has reported that PAPs can effectively promote PAP leaders’ use of research evidence in improving youths’ outcomes [12,20,10]. However, the existing literature has not yet informed the specific mechanisms of PAPs that promote PAP leaders’ use of research evidence in improving youths’ outcomes; whether and how PAP leaders’ use of research evidence evolves along the PAP life cycle; and whether PAP partners’ concordant perceptions of usefulness of their PAP has an impact on PAP leaders’ use of research evidence. Developing a conceptual framework that recognizes the PAP life cycle, and empirically identifying contexts and mechanisms of PAPs that promote PAP leaders’ use of research evidence from the PAP life cycle perspective is imperative to guide researchers and policymakers to successfully lead PAPs and foster policymakers’ use of research evidence in improving youth outcomes. This proposed project will examine the extent to which PAP development and PAP leaders’ use of research evidence can be characterized into life cycle stages, and identify PAP contexts and mechanisms that explain the progress of PAPs and PAP leaders’ use of research evidence through life cycle stages.

### Theoretical and Empirical Rationale

**Figure 1:**
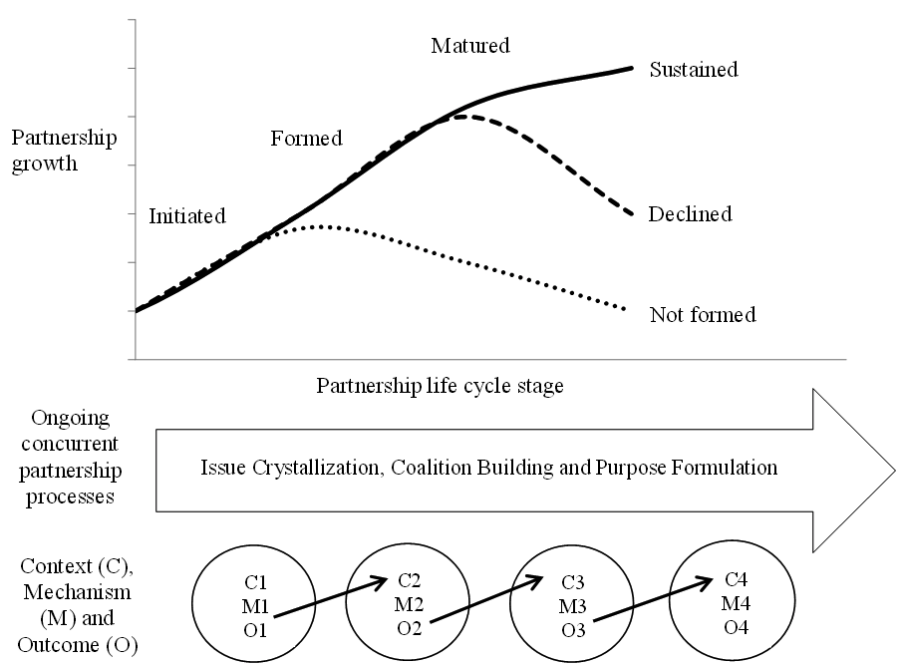
Proposed conceptual framework to understand public-academic partnerships as social partnerships transitioning through life cycle stages.

Although a number of empirical studies have helped identify specific factors that are associated with PAP success and failure, there is no research evidence that offers a framework of contexts and mechanisms that should occur at each stage of PAP development in order to maximize the chances of success and increase PAP leaders’ use of research evidence. The present project proposes an integrated framework of social partnerships, organizational life cycle, and realist evaluation perspectives to address the complex contexts and corresponding mechanisms that result in successful sustainment of PAPs and foster PAP leaders’ use of research evidence, for PAPs that serve youth in the public care sector. This project classifies the PAP life cycle stages into initiated/not initiated, formed/failed to be formed, matured/not matured, and sustained/declined (Figure 1) [21-23].

*The social partnership perspective* [7,22-28] posits that partnerships have common roots in their intended impact on some societal problems such as education, poverty, and health by building on the capabilities, resources, and expertise of each partner [25,27,28]. This perspective aligns well with PAP development, as PAPs are typically initiated to address social issues such as youth mental health and well-being. A social partnership perspective is that three key partnership processes should occur concurrently to evolve through stages such as initiation, formation, and maturity: issue crystallization; coalition building through mutual benefits and trust, top management support, convener’s role, and on-the-spot decision-making power; and purpose formulation through determining structure, goals, primary function, and process of setting agenda [20]. Although the perspective offers a compelling explanation for factors that support successful PAP initiation, formation, and maturity, the framework does not explain how PAP partnerships sustain. *The organizational life cycle perspective* [29-34] offers a longitudinal and sequenced approach to explain how PAPs grow and change over time. Organizations go through stages of birth, maturity, and decline and the goals, priorities, and definitions of organizational effectiveness differ across not only organizations but also these stages within organizations [29,34]. By applying this perspective, we can explain how PAP partnerships transition through life cycle stages and the potential presence of a sustained versus declined stage, which the social partnership perspective is missing. An integration of aspects of the organizational life cycle perspective with social partnership perspective contributes to the conceptualization of PAP progress by distinguishing specific life cycle stages relevant to the progress of organizations. *The realist-evaluation approach* [35-37] helps deepen the conceptualization of how the contexts of PAPs are involved in both their sustainability and use of research evidence. Realist evaluation focuses on three concepts—context, mechanism, and outcomes—which link together to form a context-mechanism-outcome (CMO) configuration (Figure 1). This proposed project adapts the definitions of context, mechanism, and outcome from the study of Jagosh et al [38] who used the realist evaluation approach to understand partnerships in community participatory research.

Describing context, mechanism, and outcome relationships based on the realist evaluation framework helps generate and refine an explanatory theory, which, in this case, are the theory of how PAPs evolve and what promotes or inhibits PAP leaders’ use of research evidence (Multimedia Appendix 1). The features of a realist evaluation approach can be applied to PAPs to clarify the nature and contributions of contexts and mechanisms to PAP sustainability and PAP leaders’ use of research evidence according to the PAP life cycle stage in real-world settings.

Neither social partnerships nor organizational life cycle perspectives recognize the embedding of PAPs in a system and do not offer a means to connect partnership contexts and mechanisms with PAP leaders’ use of research evidence. The realist evaluation approach is based on determining not just what approaches work, but more specifically, “what works, for whom, under what circumstances, and why and how” [35-37]. Thus, this perspective helps deepen the conceptualization of how the contexts of PAPs are involved in both their sustainability and use of research evidence.

Considering that PAP leaders’ perception of their academic partners and coalition building with their academic partners can have an impact on PAP sustainability [16,25,39], PAP sustainability and PAP leaders’ use of research evidence are likely to be associated. However, the lack of empirical findings warrants further studies. The integrated conceptual framework that includes a theory-building process will contribute to further examination of the relationship between PAP sustainability and PAP leaders’ use of research evidence. Extending the previous research [16,25,39], this proposed project seeks to examine the following aspects:

1. Do PAPs go through a life cycle of being initiated, formed, matured, and sustained?
  - Can the partnership processes (issue crystallization, coalition building, and purpose formulation) be traced through the PAP life cycle stages? If yes, how do partnership processes differ by PAP life cycle stage?
2. Which factors promote or interfere in the progression and ultimate sustainability of PAPs?
  - Do PAP partners’ perceptions of alignment between PAP purpose formulation and their own organizational purpose formulation differ by PAP life cycle stage?
  - Do PAP partners’ perceptions of PAP coalition building differ by PAP life cycle stage?
3. Are there different patterns of use of research evidence (in terms of obtaining, evaluating, and using research evidence) associated with each PAP life cycle?
4. Which PAP factors promote or inhibit PAP leaders’ use of research evidence?
  - Does PAP leaders’ use of research evidence differ by PAP partners’ perceptions of alignment between PAP purpose formulation and their own organizational purpose formulation?
  - Does PAP leaders’ use of research evidence differ by PAP partners’ perceptions of PAP coalition building?

## Methods

### Sampling and Participant Recruitment

PAPs that are comprised of at least one or more state or local county child welfare or mental health agencies, and one or more academic researchers will be eligible for inclusion. In the proposed project, mental health includes both mental health and substance abuse. The PAPs can be formed on a project/program/intervention basis or as a consortium. PAPs will be included if their aims include improving mental health and well-being outcomes for youth aged 12-25 years. If multiple academic researchers from one academic research institute are working on separate projects/programs/interventions with the same public care agency, these partnerships will be counted as multiple PAPs, as the partnerships may be in different life cycle stages with different contexts and mechanisms that affect PAP leaders’ use of research evidence differently. If one academic researcher has multiple projects with one public care agency, multiple projects will be treated as a single PAP. PAPs that were terminated before 2007 will be excluded. PAPs considered to be in an “initiating” or “failed to be formed” stage will be excluded from this recruitment stage, as members of these PAPs are likely to be difficult to identify. PAPs situated outside the United States or focusing on youth outside the United States will be also excluded. A variety of strategies including online search and contacting partners through emails and phone calls will be undertaken to recruit as many eligible PAPs as possible and collect consistent information from all PAPs. If only the PAP leaders or the academic researchers of each PAP agree to participate, we will still include the PAP in the study for supplementary data.

### Document Review and Online Survey of Public-Academic Partnership Leaders and Academic Researchers

The project team will invite all PAPs that meet the inclusion criteria to participate and concurrently conduct PAP document review and an online survey of PAP leaders and academic researchers. We will seek PAP documents that include information on PAP structure, goals, primary function, actor roles, process of setting agenda, and funding sources for the PAP processes. The document review will apply the iterative CMO configuration process of the realist evaluation approach [36,38]. An iterative CMO configuration process will be conducted, in which the PAP documents are reviewed by the project team utilizing a review protocol drafted to serve as the guiding tool in the CMO configuration of PAP life cycle based on the potential CMO of each PAP life cycle stage (Multimedia Appendix 1). PAP mechanisms and PAP leaders’ use of research evidence by life cycle stage will be detailed through the document review, and the protocol will be refined through multiple rounds of review of PAP documents.

Online survey of PAP leaders and academic researchers will collect data on partners’ PAP experience and PAP leaders’ use of research evidence. The intent is to obtain over a 75% survey response rate. PAP Survey 1: The Structured Interview for Evidence Use (SIEU) scale [16] will determine PAP leaders’ engagement level of research evidence, which refers to the frequency of using various types of sources for research evidence; PAP leaders’ ratings of the importance of evaluating the validity, reliability, and relevance of research evidence; and various factors leading PAP leaders to use/ignore research evidence in deciding to adopt a new program or intervention. The original SIEU scale items will be used. The Input scale (20 items) assesses the source of the research evidence PAP leaders obtain. The Process scale assesses how PAP leaders evaluate the research evidence obtained and includes three subscales of self-assessment for validity and reliability of research evidence (10 items), reliance on others (5 items), and self-assessment for relevance (5 items). The Output scale (20 items) assesses if PAP leaders eventually use research evidence or ignore the evidence. The measurement asks respondents to indicate responses using a Likert-type scale ranging from 1 (not at all) to 5 (all the time) for the items contained in the Input scale, and a 5-point Likert-type scale ranging from 1 (not important) to 5 (very important) for the items contained in the Process and Output scales. The SIEU shows high internal consistency reliability (α=.88) [16]. PAP Survey 2: PAP Experience (Multimedia Appendix 2) will measure PAP leaders’ and academic researchers’ most recent PAP experience through items that address issue crystallization (clear issue pursued), purpose formulation (structure, goals, primary function, and agenda setting process), coalition building (mutual benefits and trust, top management support, convener’s role, and on-the spot decision-making power); PAP partners’ perceptions of their PAP life cycle stage; and PAP leaders’ use of research evidence. The online surveys will be built in and administered through the Research Electronic Data Capture, a secure Web-based data collection tool that includes data entry forms and Web surveying features.

### Analysis

For the document review, the principal investigator and a research assistant will first independently classify the life cycle for each PAP (formed, but not yet matured; matured, but not reached a sustained stage yet; and sustained/declined) by applying the potential CMO of each PAP life cycle stage (Multimedia Appendix 1) and then review the classification of PAPs until a consensus is reached on the classification. Information on PAPs such as partnership structure, goals, and primary function can vary depending on the available documentation. For missing or incomplete data during the document review process, the project team will follow-up with academic researchers and PAP leaders through emails and phone calls to request and obtain the missing information. The online survey data will also complement the missing data from the document review as the domains of the survey questionnaire are consistent with those of the document review protocol.

Concurrently, the online survey data on PAP leaders’ use of research evidence and experience with PAPs will be analyzed in relation to the CMO configuration process. For the online survey, reliability of the SIEU will be calculated by using the Cronbach α internal consistency for each of the subscales and the total scale. We will descriptively test for mean differences in PAP leaders’ engagement level of research evidence by (1) PAP partners’ rating of the level of alignment between PAP structure, goals, primary function, and process of setting agenda and their organizational structure, goals, primary function, and process of setting agenda; (2) PAP partners’ rating of the level of mutual benefit and trust, top management support, convener’s role, and on-the-spot decision-making power; and (3) PAP life cycle stage (formed, but not yet matured; matured, but not reached a sustained stage yet; and sustained/declined). Concordance levels of PAP leaders’ and academic researchers’ perceptions of PAP contexts and mechanisms will be calculated according to the PAP life cycle stage using Cohen κ coefficients and McNemar test [40]. Pearson product-moment correlations of the concordance levels and SIEU subscale and total scores will test the relationship between the concordance of partner’s perceptions and PAP leaders’ use of research evidence. The potential CMO of each PAP life cycle stage (Multimedia Appendix 1) will be refined through multiple rounds of review of PAP documents and quantitative data for the development of a middle-range theory.

## Results

Fifty-three eligible PAPs have been identified, document review of 20 PAPs have been conducted, and 16 PAP researchers have been reached out for additional information. The principal investigator and the research assistant are in the process of classifying the life cycle for each PAP based on the document review by applying the potential CMO of each PAP life cycle stage (Multimedia Appendix 1). The classification of PAPs will be continued until consensus is reached on the classification. Concurrently, the project team is recruiting PAP leaders and academic researchers who will participate in the online survey and will conduct analysis of CMO PAP life cycle stages and its relationship to PAP leaders’ use of research evidence.

## Discussion

The proposed project is expected to help policymakers and researchers gain a deeper knowledge of the contexts and mechanisms for each PAP life cycle stage in order to optimize PAP leaders’ use of research evidence in achieving positive youth outcomes. Although we will focus on youth mental health and well-being, our findings are likely to be relevant to other vulnerable populations. Future studies should include PAPs in an “initiating” or “failed to be formed” stages, as the PAPs are likely to provide valuable learning about attempted partnerships.

## Appendix

Multimedia Appendix 1 Potential Context, Mechanism and Outcome (CMO) of each Public-Academic Partnership (PAP) life cycle stage.

Multimedia Appendix 2 Public-Academic Partnership (PAP) Survey Questionnaire.

Multimedia Appendix 3 Peer-reviewer report from the William T Grant Foundation.

## Abbreviations

CMO: context-mechanism-outcome
PAP: public-academic partnership
SIEU: Structured Interview for Evidence Use
