# Supplementary material for: Optimizing the Impact of Public-Academic Partnerships in Fostering Policymakers’ Use of Research Evidence: Proposal to Test a Conceptual Framework
Source: JMIR Res Protoc. 2019 May 24;8(5):e14382. doi: 10.2196/14382 (PMC6555116; doi:10.2196/14382)
Supplement: Multimedia Appendix 1 [file resprot_v8i5e14382_app1.pdf]

Potential Context, Mechanism and Outcome (CMO) of Each Public-Academic Partnership (PAP) Life Cycle Stage

	PAP Life Cycle Stages			
**Potential PAP Contexts, Mechanisms and Outcomes (CMO) to be explored and identified**	**Initiation /Not Initiated**	**Formed/Not Formed**	**Matured/Not Matured and Declined**	**Sustained/Declined**
**Contexts: Partnership processes**				
Issue crystallization context - Specific environmental forces:<br>• Legal system or mandate<br>• Existing social networks which introduce potential partnership members to one another and to mutual concerns and benefits<br>• Common vision or a common understanding within a community about an issue and how it should be handled<br>• Crisis, which directs the energies of potential partners toward a specific problem<br>• Visionary leadership, which is embodied in an individual as opposed to a group	*Specific environmental forces identified*	*Specific environmental forces will bring partners together and result in a PAP.*	*Environmental forces will be continuously scanned.*	*Specific environmental forces will be continuously scanned and new environmental forces will be identified.*
Issue crystallization context – A clear common issue	*A clear issue that will bring partners together and result in PAP initiation will be identified.*	*A clear issue to form a partnership will be agreed and documented.*	*A new issue to focus will be identified.*	*A new issue to focus will have resulted in a purpose reformulation.*
Coalition building contexts – Key actors' involvement:<br>• On-the-spot decisionmaking power<br>• Support of top management<br>• A "convener," an individual or a group with sufficient authority and status to bring potential partners into the partnership forum	*Key actors will be identified.*	*Key actors will agree and participate in the PAP processes.*	*Key actors will keep playing their role in the PAP processes. Conflicts might rise and the key actors will appropriately manage the conflicts.*	*Key actors will continuously support the PAP processes.*
Purpose formulation contexts<br>• Structure:<br>▪ Research-dominant partnership or equally responsible for leadership<br>▪ Administrative, communication and decisionmaking structure;<br>• Goals and primary function:<br>▪ Generate knowledge related to the development	*PAP structure, goals and primary function, and process of setting agenda will be discussed.*	*Detailed PAP structure, goals and primary function, and process of setting agenda will be agreed and documented by partners.*	*Detailed PAP structure, goals and primary function, and process of setting agenda set by the partners will be built-in as routine partnership processes.*	*PAPs are likely to go through ongoing purpose reformulation.*

	PAP Life Cycle Stages			
**Potential PAP Contexts, Mechanisms and Outcomes (CMO) to be explored and identified**	**Initiation /Not Initiated**	**Formed/Not Formed**	**Matured/Not Matured and Declined**	**Sustained/Declined**
of evidence-based/evidence-informed policymaking and practices<br>▪ Implementation of evidence-based/evidence-informed policy and practices<br>▪ Generalize practices to a larger population of children and adolescents in need of services<br>▪ Generate and disseminate knowledge related to the implementation of evidence-based/evidence-informed policy and practices<br>▪ Offering technical assistance, training for professionals and/or program evaluation in improving service quality and outcomes<br>• Process of setting agenda:<br>▪ Research agendas are driven both by the needs of PAP policymakers to deliver high quality services and/or by the desire of researchers to use the community as a "natural laboratory" for developing, testing, and implementing evidence-based/evidence-informed policymaking and practices in the public care sector<br>▪ Participatory decisionmaking vs. top-down decision making			*PAPs will have achieved the goals set in the formation stage and are ready for purpose reformulation.*	
• *"Hooks" as a context to incentivize partnership members* to go through an iterative pattern of purpose development leading to implementation that will crystallize new issues, further develop the coalition, and formulate new purposes	*Not yet utilized*	*May be used*	*In use of "hooks"*	*Well utilized "hooks"*
• *Funding as a context to support partnership processes*	*Seeking funding*	*Funding secured*	*Funding continued and new funding identified*	*Continuous or new funding secured based on purpose reformulation*
**Mechanisms: PAP partners' perception**				
PAP partners' perceptions of partnership processes:<br>• Interdependence	*Partners will begin developing*	*The previous PAP CMOs as well as the*	*The previous PAP CMOs as well as the*	*Partners' perceptions are likely to change as*

	PAP Life Cycle Stages			
**Potential PAP Contexts, Mechanisms and Outcomes (CMO) to be explored and identified**	**Initiation /Not Initiated**	**Formed/Not Formed**	**Matured/Not Matured and Declined**	**Sustained/Declined**
• Perceived mutual benefits<br>• Salience of the issue of focus<br>• Balanced power<br>• Top management support<br>• Convener’s role<br>• On-the-spot decisionmaking power	*perceptions in some of the aspects (i.e., salience of the issue of focus and top management support) of PAP processes.*	*current PAP contexts are likely to have an impact on the partners’ perceptions in this stage. Partners’ perceptions will develop in all aspects of the partnership processes.*	*current PAP contexts are likely to result in clear perceptions of the partnership processes.*	*the PAP goes through purpose reformulation.*
PAP partners’ perceptions of alignment between PAP and own organization in the following processes:<br>• PAP structures<br>• Goals and primary function<br>• Process of setting agenda	*Partners will be learning each other’s structure, goals and primary function, and process of setting agenda.*	*As partners formalize PAP structure, goals and primary function, and process of setting agenda, the partners are likely to identify similarities and differences between the PAP structure and processes and those of their organization.*	*PAP structure and processes will be built in as routine practice, and thus, partners are likely to be clearly aware of similarities and differences in the alignment between the PAP structure and processes and those of their organization.*	*The partners are likely to attempt to align the PAP processes with partner organizations’ structure, goals and processes.*
**Outcomes to be identified:**<br>• PAP life cycle stage	*PAP initiated vs. not initiated*	*PAP formed vs. PAP failed to be formed*	*PAP matured vs. PAP not matured and declined*	*PAP sustained vs. PAP declined*
**Outcomes to be identified:**<br>• PAP leaders’ use of research evidence	*To be explored based on the CMOs in this stage*	*To be explored based on the CMOs in both the previous stage and this stage*	*To be explored based on the CMOs in both the previous stages and this stage*	*To be explored based on the CMOs in both the previous stages and this stage*
