# Supplementary material for: Optimizing the Impact of Public-Academic Partnerships in Fostering Policymakers’ Use of Research Evidence: Proposal to Test a Conceptual Framework
Source: JMIR Res Protoc. 2019 May 24;8(5):e14382. doi: 10.2196/14382 (PMC6555116; doi:10.2196/14382)
Supplement: Multimedia Appendix 2 [file resprot_v8i5e14382_app2.pdf]

Public-Academic Partnership (PAP) Experience Survey for Researchers and Public Care Agency Leaders

You are being invited to participate in a research study titled Optimizing the Impact of Public-Academic Partnerships in Fostering Policymakers' Use of Research Evidence. This study is being done by Christina Kang-Yi, Ph.D., Research Assistant Professor, from the Center for Mental Health at the University of Pennsylvania Perelman School of Medicine. The Penn Center for Mental Health, the more than 30-year-old multi-disciplinary center connects research and evaluation findings to policy decisions and to delivery and implementation of services to improve the lives of people with psychiatric and developmental disabilities.

You are selected to participate in this study because of your central role in improving service delivery and decision making to promote youth mental health and wellbeing. The purpose of this study is to learn whether and how public-academic partnerships support public care agency leaders in improving policy and practice for youths. We would like to ask you about your experience of partnering with public care agencies to improve youth mental health and/or well-being. In our study, public-academic partnerships (PAPs) are defined as collaboration between public care agency leaders and academic researchers/institutes in framing community problems, designing programs, implementing new initiatives and evidence-based practice, evaluating programs, and/or making policy decisions to promote youth mental health and well-being. Many PAPs are formed to improve youth mental health and well-being. Sharing your experience of PAP is very important to us because of your central role in improving youth service delivery and policymaking. The survey will take you approximately 15-20 minutes to complete.

You may not directly benefit from this research; however, we hope that your participation in the study may contribute to improving youth mental health and wellbeing served by public care agencies.

We believe that there are no known risks associated with this research study; however, as with any online related activity the risk of a breach of confidentiality is always possible. To the best of our ability, your answers in this study will remain confidential. We will minimize any risks administering and collecting the survey through the Research Electronic Data Capture (REDCap), a secure web-based data collection tool that includes data entry forms and web surveying features. Only Dr. Kang-Yi and research assistants who directly collect and analyze the survey data will have access to the REDCap project folder. Downloaded information for the data analysis will be stored in a password-protected secure data server.

Your participation in this study is completely voluntary and you have 14 days to complete this survey and withdraw at any time.

If you feel you would like assistance, please contact Dr. Christina Kang-Yi, the Principal Investigator of this study at the University of Pennsylvania Perelman School of Medicine (by phone: ***-***-****) and email: *****.

If you have questions about this project or if you have a research-related problem, you may contact Dr. Christina Kang-Yi, the Principal Investigator. If you have any questions concerning your rights as a research subject, you may contact the Office of Regulatory Affairs by calling ***-***-****.

By clicking "I agree" below you are indicating that you are at least 18 years old, have read and understood this consent form and agree to participate in this online survey.	◯ I agree<br>◯ I do not agree

1	Are you currently involved in a public-academic partnership that aims to improve mental health and/or well-being of youth aged 12-25 years?	◯ Yes<br>◯ No
	Comments:	______________________
1a	If you answered "no," have you ever been involved in a public-academic partnership to improve mental health and/or well-being of youth aged 12-25 years during the past ten years?	◯ Yes<br>◯ No
	Comments:	______________________

Please try to remember the most recent public-academic partnership you are (were) involved and answer the questions below.

Please list the project name for your partnership with the public care agency.

____

What month and year was the partnership formed? (MM/YYYY)

____

Comments:

____

Is the partnership still ongoing?

- ◯ Yes
- ◯ No

Comments:

____

If you answered "NO" what month and year was the partnership terminated? (MM/YYYY)

____

Comments:

____

On which youth groups does/did your partnership focus? Check all that apply.

- ☐ Youth with psychiatric disorders
- ☐ Youth in foster care
- ☐ Youth with child welfare system involvement
- ☐ Youth with juvenile system involvement
- ☐ Immigrant youth
- ☐ Racial/ethnic minority youth
- ☐ Homeless youth
- ☐ LGBT youth
- ☐ Youth with behavioral problems
- ☐ Youth with substance abuse problems
- ☐ Other

Comments:

____

If you selected "Other," please list all youth groups served.

____

Comments:

____

What aspects of youth service delivery or policymaking does/did your partnership aim to improve? Check all that apply.	☐ Case management<br>☐ Mental/behavioral health treatment<br>☐ Advocacy<br>☐ Health disparities/health equity<br>☐ Legal support<br>☐ Social services<br>☐ Youth mentoring program<br>☐ School-based intervention<br>☐ Family engagement<br>☐ Caregiver support<br>☐ Peer support<br>☐ Other
Comments:	__________________________________
If you selected "Other," please list all that apply.	____
Comments:	__________________________________
What is/was the main purpose of the partnership? Check all that apply.	☐ Information sharing<br>☐ Initiating new program/project<br>☐ Implementing evidence-based practice/ fidelity measures<br>☐ Program and/or policy evaluation<br>☐ Prioritizing key issues to focus<br>☐ Consulting on making policy decisions<br>☐ Streamlining research activities that involve youth and families<br>☐ Consulting on hiring staff<br>☐ Advocacy<br>☐ Dissemination of research/program evaluation evidence<br>☐ Professional training<br>☐ Conduct research
Comments:	__________________________________
If you checked "Implementing evidence-based practice/fidelity measure," has/had the fidelity measure been used in implementing evidence-based practice through the partnership?	◯ Yes<br>◯ No
Comments:	__________________________________
How is/was fidelity measured? Check all that apply.	☐ On-site observer rated<br>☐ Phone administered by implementer<br>☐ Self-reported and implementer scored
Comments:	__________________________________

Has/had the use of fidelity measures been successfully implemented with regard to completing the measure?	◯ Yes<br>◯ No
Comments:	__________________
Has/had the use of fidelity measures been successfully implemented with regard to offering technical assistance when fidelity is low?	◯ Yes<br>◯ No
Comments:	__________________
Has/had the use of fidelity measures successfully improved youth outcomes?	◯ Yes<br>◯ No
Comments:	__________________
Who initiated the partnership?	◯ Researcher(s)<br>◯ Public care agency<br>◯ County government<br>◯ State government<br>◯ Other
If you selected "Other," please specify:	______________________
Comments:	__________________

What is/was the funding source(s) for the partnership activities? Check all that apply for both current and past funding source(s).

	Current	Past	N/A	Don't know
Medicaid	☐	☐	☐	☐
County discretionary funding	☐	☐	☐	☐
City grant	☐	☐	☐	☐
Regional grant	☐	☐	☐	☐
Federal or state government grant	☐	☐	☐	☐
Non-profit foundation grant	☐	☐	☐	☐
For-profit corporation funding	☐	☐	☐	☐
Agency discretionary funds	☐	☐	☐	☐
United Way	☐	☐	☐	☐
Other	☐	☐	☐	☐

If you selected "other," please list specific funding sources and indicate "current" or "past."

____

Comments:

____

How many members are/were involved in the partnership?

	0	1	2	3	4	5	6	7	8	9	10 or more
Researchers	○	○	○	○	○	○	○	○	○	○	○
Executive directors	○	○	○	○	○	○	○	○	○	○	○
Project directors/managers	○	○	○	○	○	○	○	○	○	○	○
Postdoctoral fellows	○	○	○	○	○	○	○	○	○	○	○
Student assistants	○	○	○	○	○	○	○	○	○	○	○
Research coordinators	○	○	○	○	○	○	○	○	○	○	○
Other staff	○	○	○	○	○	○	○	○	○	○	○
Service recipients	○	○	○	○	○	○	○	○	○	○	○
Administrative assistants	○	○	○	○	○	○	○	○	○	○	○

Comments: ______________________________

How would you rate the current partnership in the following domains?

Does the partnership have a clear issue to focus in improving youth outcomes?	◯ Yes<br>◯ Not yet<br>◯ Used to have
Comments:	__________________________

Does/did the partnership have specific governance and/or operational process documented?

	Yes	No	Not yet	Used to have
Governance	◯	◯	◯	◯
Operational process	◯	◯	◯	◯

Comments: __________________________________

How well is/was the partnership structure aligned with your organizational structure? Please focus on the administrative, communication and decision-making structure and dominance of research as you answer this question.

◯ Not at all
◯ Very little
◯ Fairly well
◯ Quite Well
◯ Very well
◯ Perfectly

Comments: __________________________________

How well are/were the partnership goals aligned with your organizational goals?

◯ Not at all
◯ Very little
◯ Fairly well
◯ Quite Well
◯ Very well
◯ Perfectly

Comments: __________________________________

How well is/was the partnership's primary function aligned with your organization's primary function?

◯ Not at all
◯ Very little
◯ Fairly well
◯ Quite Well
◯ Very well
◯ Perfectly

Comments: __________________________________

How well is/was the partnership's process of setting an agenda aligned with your organization's process of setting an agenda in the following areas?

	Not at all	Very little	Fairly well	Quite Well	Very well	Perfectly
Agenda-setting process is driven by the needs of policymakers to deliver high quality services	◯	◯	◯	◯	◯	◯
Agenda-setting process is driven by the desire of researchers to use the community as a "natural laboratory" for developing, testing, and implementing evidence-based/evidence-informed policymaking and practices in the public care sector	◯	◯	◯	◯	◯	◯
Agenda-setting process is driven by participatory decision-making	◯	◯	◯	◯	◯	◯
Agenda-setting process is driven by top-down decision-making	◯	◯	◯	◯	◯	◯

Comments: ______________________________

Does/did the partnership have clear academic researcher and public agency leader representation and roles?

- ◯ Never
- ◯ Rarely
- ◯ Occasionally
- ◯ Frequently
- ◯ Very frequently
- ◯ Always
- ◯ Used to have clear representation and roles

Comments: ______________________________

Does/did the partnership have a person playing the role of gathering people together to carry out partnership processes such as crystallizing an issue, building partnership coalition and setting agenda?

- ◯ Yes
- ◯ No
- ◯ Used to have

Comments: ______________________________

If you answered "yes" or "used to have," what is/was the job title of the person with this role in the public care agency? If more than one person play the convening role, list all job titles. ______________________________

Comments: ______________________________

If you answered "yes" or "used to have," what is/was the job title of the person with this role in the research institute? If more than one person play the convening role, list all job titles.

____

Comments:

____

Who has/had the on-the-spot decision-making power in your partnership? List the job title(s) of the decision maker(s) in the public care agency.

____

Comments:

____

Who has/had the on-the-spot decisionmaking power in your partnership? List the job title(s) of the decision maker(s) in the research institute.

____

Comments:

____

Does/did the partnership pursue mutual benefits in setting agenda?

- ◯ Never
- ◯ Rarely
- ◯ Occasionally
- ◯ Frequently
- ◯ Very frequently
- ◯ Always
- ◯ Used to pursue mutual benefit

Comments:

____

How does/did the top management from the research institute support the partnership process?

	Never	Rarely	Occasion-ally	Frequentl y	Very frequently	Always	Used to	N/A
Attend(ed) partnership meetings regularly	○	○	○	○	○	○	○	○
Obtain(ed) funding for partnership sustainability	○	○	○	○	○	○	○	○
Provide(d) input in planning and agenda setting	○	○	○	○	○	○	○	○
Communicate(d) with stakeholders to achieve the partnership goals and support activities	○	○	○	○	○	○	○	○
Disseminate(d) outcomes based on the partnership activities	○	○	○	○	○	○	○	○
Recruit(ed) and designate(d) partnership members	○	○	○	○	○	○	○	○
Provide(d) resources (e.g., staff space for meetings, etc.)	○	○	○	○	○	○	○	○

How does/did the top management from the public care agency support the partnership process?

	Never	Rarely	Occasion-ally	Frequently	Very frequently	Always	Used to	N/A
Attend(ed) partnership meetings regularly	◯	◯	◯	◯	◯	◯	◯	◯
Obtain(ed) funding for partnership sustainability	◯	◯	◯	◯	◯	◯	◯	◯
Provide(d) input in planning and agenda setting	◯	◯	◯	◯	◯	◯	◯	◯
Communicate(d) with stakeholders to achieve the partnership goals and support activities	◯	◯	◯	◯	◯	◯	◯	◯
Disseminate(d) outcomes based on the partnership activities	◯	◯	◯	◯	◯	◯	◯	◯
Recruit(ed) and designate(d) partnership members	◯	◯	◯	◯	◯	◯	◯	◯
Provide(d) resources (e.g., staff space for meetings, etc.)	◯	◯	◯	◯	◯	◯	◯	◯

Comments: __________________________________

How often does/did the PAP membership change (e.g., new members joining the PAP or replacing other PAP members)?

	Biannually	Annually	Every few years	Never
Within the research institute	◯	◯	◯	◯
Within the public care agency	◯	◯	◯	◯

Comments: ______________________________________

Has/had there been a new leader of the PAP?

	Biannually	Annually	Every few years	Never
Within the research institute	○	○	○	○
Within the public care agency	○	○	○	○

Comments:

____

Have/had you experienced conflict(s) in the partnership process?	◯ Yes<br>◯ No
Comments:	__________________
Do/did the partnership members know how to appropriately handle conflicts?	◯ Yes<br>◯ No
Comments:	__________________
Have/had you experienced challenges or unexpected events in the partnership process?	◯ Yes<br>◯ No
Comments:	__________________
Do/did the partnership members know how to appropriately handle challenges or unexpected events?	◯ Yes<br>◯ No
Comments:	__________________
Has/had the partnership resulted in identifying another issue (e.g., service delivery, state policy change, evidence-based practice, and information dissemination) to focus on?	◯ Yes<br>◯ No
Comments:	__________________

	Yes	No
Has/had the new issue led the partnership to reformulate its structure (e.g., membership and frequency of meetings)?	◯	◯
Has/had the new issue led the partnership to reformulate its goals and primary function?	◯	◯
Has/had the new issue led the partnership to reformulate its agenda setting process?	◯	◯

Comments:	__________________

What is/was both parties' overall level of trust?

	High	Moderate	Low	None	Used to have high level of trust
Level of trust the researcher has/had for the PAP leader	◯	◯	◯	◯	◯
Level of trust the PAP leader has/had for the researcher	◯	◯	◯	◯	◯

Comments: ______________________________

Question	Response
Has the partnership encouraged the degree to which PAP leaders (public care agency leaders) use research evidence?	◯ Yes<br>◯ No<br>◯ Don't know
Comments:	__________________________________
Has the partnership discouraged the degree to which PAP leaders (public care agency leaders) use research evidence?	◯ Yes<br>◯ No<br>◯ Don't know
Comments:	__________________________________
Of the following partnership development stages, which stage do you categorize your partnership?	◯ Formed, but not yet matured<br>◯ Matured, but not reached a sustained stage yet<br>◯ Sustained<br>◯ Declining<br>◯ Terminated
Comments:	__________________________________
Does/did the partnership have a process of evaluating the outcomes aimed to be achieved by the partnership? Check all that apply.	☐ Evaluation planning<br>☐ Evaluation design<br>☐ Data collection/obtainment and analysis<br>☐ Dissemination of findings<br>☐ Other
Comments:	__________________________________
If you selected "other," list specific process.	____
Comments:	__________________________________
Would you be willing to share the following types of reports on the outcomes resulted in by your PAP? Check all that apply.	☐ Journal articles<br>☐ Annual reports<br>☐ Technical reports<br>☐ Infographics<br>☐ Policy briefs<br>☐ Presentation slides<br>☐ Government reports issued by county, state, etc.<br>☐ Other<br>☐ No
Comments:	__________________________________
If you selected "other," list the type of evidence.	__________________________________

Comments: ________________________________

What is your job title? __________________________________

Comments: __________________________________

What role(s) do/did you play within the PAP? ____

Comments: __________________________________

How many years of experience do you have in this field? __________________________________

Comments: __________________________________

How long (in years) have you been a part of your current organization? __________________________________

Comments: __________________________________

How long have/had you been involved with the current PAP? __________________________________

Comments: __________________________________

What is the total number of PAPs you have participated in, including the current PAP? __________________________________

Comments: __________________________________

What is your age (in years)? __________________________________

Comments: __________________________________

What is the highest level of education you have completed?

- ◯ High school diploma
- ◯ Bachelor's degree
- ◯ Master's degree
- ◯ Doctoral degree
- ◯ Professional degree

Comments: __________________________________

What is your racial/ethnic identification?	◯ Hispanic/Latino<br>◯ White<br>◯ African-American/Black<br>◯ Asian/Pacific Islander<br>◯ Native American/American Indian<br>◯ Multi-racial<br>◯ Other (Please specify)
Comments:	__________________________
If you selected "other," please describe your racial/ethnic identification:	________________________________
Comments:	__________________________
What is your gender identification?	◯ Male<br>◯ Female<br>◯ Prefer to self-describe
Comments:	__________________________
If you prefer to self-describe, please include your response here.	__________________________
Comments:	__________________________

If you are willing to share the materials with us, our project team will follow up with you to obtain the reports. Please provide your name, email address and phone number.

Name ________________________________

Email address ________________________________

Phone number ________________________________

Thank you for your participation!
