# Supplementary material for: Optimizing the Impact of Public-Academic Partnerships in Fostering Policymakers’ Use of Research Evidence: Proposal to Test a Conceptual Framework
Source: JMIR Res Protoc. 2019 May 24;8(5):e14382. doi: 10.2196/14382 (PMC6555116; doi:10.2196/14382)
Supplement: Multimedia Appendix 3 [file resprot_v8i5e14382_app3.pdf]

December 19, 2017

Christina Kang-Yi, Ph.D.
University of Pennsylvania
Perelman School of Medicine
3535 Market Street, 3rd Fl.
Philadelphia, PA 19104

Application ID#188269
Optimizing the Impact of Public-Academic Partnerships in Fostering Policymakers' Use of Research Evidence

Dear Dr. Kang-Yi:

Thank you for your revised application to examine how public-academic partnerships promote the use of research evidence by state and local child welfare and behavioral health services. We recently received three external reviews of your proposal (attached) as well as input from senior staff and all agreed that the proposal addressed many of the earlier concerns. We are pleased to offer you an opportunity to respond to the concerns raised.

A brief description of the Foundation's review procedures may be helpful. We use a scientific peer review process. First, we obtain external reviews. We attempt to select consultants whose expertise is relevant to your proposal and who bring different perspectives to the review. These reviews reflect the reactions of experts in the field and do not necessarily represent the Foundation's views. As a second step, at least two senior staff members critique the proposal and reviews.

At this stage of the process, select applicants have an opportunity to respond to reviewers' and staff members' comments and offer adjustments to strengthen their proposals. Although the reviews include many positive comments, we ask applicants to focus on addressing the critical concerns. Competition for funding is tight, and thus even favorably reviewed proposals undergo much additional scrutiny in our final funding decisions.

The following questions and recommendations are offered to strengthen the study's contribution to the field.

Sampling

Reviewer 2 suggests sampling public-academic partnerships with some consideration of youth outcomes. This reviewer argues that the project would be more strongly aligned with the Foundation's interest in improving the use of research evidence if the study also examined the link between child outcomes and the quality and developmental stage of the partnership. Please discuss the appropriateness and feasibility of this recommendation or, as an alternative, whether it might be possible to link the quality of services delivered to various dimensions of the partnership.

Reviewer 3 raises two issues that may have implications for sample recruitment. First, this reviewer worries that you may be underestimating the obstacles to recruitment. Second, they are concerned that variability in researchers' roles and commitment to partnerships may result in the selection of highly variable collaborations. Please engage with each of these concerns and describe your plans for addressing these challenges.

Assessing the Use of Research Evidence

The proposal pursues interesting questions about what contexts and mechanisms may facilitate partnerships that are effective in promoting the use of research evidence. The success of the study hinges on strong assessments of partnerships and the use of research evidence. The proposal does an excellent job assessing public-academic partnerships. We encourage you to revisit the opportunities to assess research use—in the interviews, document reviews, and site visits—and further consider the extent to which the proposed work will generate valid and nuanced measures of research use.

Lastly, please consider reviewers' suggestions for reducing the budget.

Thank you again for your responsiveness to earlier reviews. We appreciate the considerable time you have invested in this application.

Kimberly DuMont, Ph.D.
Senior Program Officer
KD:nrt

RESEARCH GRANTS EXTERNAL REVIEW

Review #1

**Foundation Focus Area**	Use of Research Evidence
**EGID**	188269
**Principal Investigator:**	Christina Kang-Yi
**Organization:**	University of Pennsylvania

THEORETICAL AND EMPIRICAL RATIONALE

1. **Fit with the Foundation's Interests. Comment on the proposal's fit with the Foundation's interest in improving the use of research evidence. We support research to do any of the following: 1) identify, create, and test the structural and social conditions that foster more routine and constructive uses of existing research evidence; 2) identify, create, and test the incentives, structures, and relationships that facilitate the production of new research evidence that responds to decision makers' needs; and 3) investigate whether and under what conditions using high- quality research improves decision making and youth outcomes.**

   The proposal fits with the Foundation's interest in improving the use of research evidence.

2. **Does the proposed work reflect a mastery of prior theory and empirical work on the use of research evidence? Would it advance that field in significant ways?**

   The proposal does a very good job of summarizing and synthesizing relevant theoretical and empirical literatures on the use of research evidence by public-academic partnerships (PAPS). The proposal makes a strong case for using the organizational life cycle perspective and social partnerships framework to better understand the development and success of PAPs. This study has potential to significantly advance knowledge about public-academic partnerships.

3. **Are the policy or practice issue(s) sensible ones to study? Is there a compelling rationale that research evidence plays any role (i.e., conceptual, instrumental, political, etc.) in those policy or practice issues?**

   The proposed project aims to understand the role of PAPs in engaging state and local policymakers in the use of research evidence as they frame problems, design and implement programs, and make policy decisions for youth involved with public child welfare and behavioral health systems. There have been important policy developments concerning the policies and programs directed towards these youth in recent years and research has played a role in those developments. As the proposal points out, state and local system leaders have developed a greater interest in identifying program effects and cost efficiency, in deciding whether to adopt a program, in evaluating information from experts or community members,

and in identifying programs that will meet the information needs of these leaders. The revised proposal's focus on the broad population of youth involved in behavioral health and child welfare services rather than the earlier more limited focus on transition-age foster youth provides for a more compelling (and more feasible) study.

4. **Does the applicant propose clear and compelling research questions and/or hypotheses?**

The research questions are clearly stated, are important, and the findings are likely to be relevant to PAPs in general.

RESEARCH DESIGN, METHODS, AND ANALYSES

1. Research Design and Methods

1a. Comment on the strength of the research design and sampling plan for addressing the research questions and/or hypotheses. Consider the sampling of cases, sites, individuals, documents, and so on.

The study design appears to be appropriate for the study's research questions and addresses my concern raised about the feasibility of the study originally proposed by the PI. It appears reasonable to believe that the study team can identify the PAPs that they will need to carry out the study.

1b. Comment on the data collection plan, including the strength of the data collection tools (i.e., interview or observation protocols, coding schemes, measures, etc.) and the plans to field those tools.

The proposal does an admirable job of describing and justifying the measures the PI and her collaborators plan to use. The measures and data collection plan flow logically from the study's research questions and the theoretical frameworks informing the proposed study.

1c. If the applicant proposes measurement development work, comment on (i) the importance of the construct, (ii) the utility of the measure for the field, (iii) the rigor of the measurement work, and (iv) the team's measurement expertise.

The proposed study does not focus on measurement development.

1d. Comment on the feasibility of the methods and data collection plan. Many applicants propose to collect sensitive data from policymakers and practitioners. Are you persuaded that the team can successfully collect that data? For example, do they provide evidence that they have successfully done so in the past? Do they have convincing strategies for maximizing response rates and gaining access to respondents, meetings, and documents?

The feasibility of the study is much improved over the original study given the revised focus on a broader population of youth and hence a larger number of PAPs to potentially involve in the study. The informants from the PAPs (agency leadership and researchers) are likely to be interested in participating in such a study and the data collection called for does not appear particularly burdensome. The proposed data collection activities appear feasible.

2. Data Analysis and Interpretation

2a. Comment on the sophistication and rigor of the analysis plan. Consider whether the team demonstrates an understanding of the strengths and limits of various analytic techniques.

The analysis plan is appropriate for the research questions and the data to be collected. The study team, including its advisors, has experience working with similar data and methods.

3. Do you have other comments on the significance or rigor of the proposed work?

No.

ADDITIONAL CONSIDERATIONS

1. Is the budget appropriate? Does it reflect a cost-effective use of the Foundation's limited resources? Describe any concerns.

The budget appears to be appropriate given the scope of the proposed work.

2. Is the project team adequately staffed to successfully carry out the project? If not, what staffing or expertise is needed? (Please note that the Foundation discourages large advisory committees. Each proposed advisor should serve a specific and significant need for the project.)

The research team has expertise in all of the substantive and methodological domains that the proposed work relies on. The revised scope of work and the expertise of project advisors address my earlier concern about a lack of expertise on transition-age foster youth.

RESEARCH GRANTS EXTERNAL REVIEW

Review #2

**Foundation Focus Area**	Use of Research Evidence
**EGID**	188269
**Principal Investigator:**	Christina Kang-Yi
**Organization:**	University of Pennsylvania

THEORETICAL AND EMPIRICAL RATIONALE

1. **Fit with the Foundation's Interests. Comment on the proposal's fit with the Foundation's interest in improving the use of research evidence. We support research to do any of the following: 1) identify, create, and test the structural and social conditions that foster more routine and constructive uses of existing research evidence; 2) identify, create, and test the incentives, structures, and relationships that facilitate the production of new research evidence that responds to decision makers' needs; and 3) investigate whether and under what conditions using high- quality research improves decision making and youth outcomes.**

The proposed study aims to develop a conceptual framework within which to understand dynamic and complex public-academic partnerships (PAPs). It is concerned to know what factors sustain PAPs and how they may promote use of research evidence. In the proposal, alignment with the Foundation's interests is described in the following way: "The proposed study aligns with the William T. Grant Foundation's objective of gaining deeper knowledge of partnerships between researchers and policymakers to cultivate use of research evidence among policymakers in achieving positive youth outcomes." This would seem to suggest alignment with Foundation interest 3): "investigate whether and under what conditions using high-quality research improves decision making and youth outcomes."

Youth outcomes are typically mentioned in the proposal within the following recurring prepositional phrase: "to cultivate use of research evidence among policymakers in achieving positive youth outcomes." Youth outcomes per se are not measured. Thus, to agree that this proposal fits with #3, above, one has to assume that public-academic partnerships are effective in promoting use of research evidence and that use of research evidence is related to improved youth outcomes or that it is ok for a proposal to fit a portion of each stated interest.

This assumption seems reasonable given additional WTG guidance documents which state, "...we welcome studies that examine how and under what conditions efforts to develop trusting relationships, sustained partnerships, and other processes connect the research and policy and practice worlds in ways that benefit decision making and youth outcomes. In sum, we are looking for projects that systematically study how to create the conditions that foster the

production of relevant research and its routine use, as well as studies that examine what happens when research is used."

In my opinion, this proposal aligns exquisitely with the Foundation's interest in studies that "systematically study how to create the conditions that foster... routine use of research evidence."

2. **Does the proposed work reflect a mastery of prior theory and empirical work on the use of research evidence? Would it advance that field in significant ways?**

The proposed work reflects a significant personal and academic engagement with the available literature on public-academic partnerships—both in child welfare and mental health systems. The work demonstrates mastery of prior theory on PAPs, including both social partnership and organizational life cycle theory. It seeks to extend theory on PAPs by developing a conceptual framework that systematically identifies contexts and mechanisms of PAPs that promote PAP leaders' use of research evidence from the PAP life cycle perspective.

Given the explicit wish of the Foundation to examine "the use of research evidence by state and local decision makers, intermediaries, and middle management" and moreover, this proposed study's aim to produce "a middle-range theory of optimizing public-academic partnerships in fostering policymakers' use of research evidence," the proposed work stands to advance the field in significant ways.

Study results are described as "a 'lessons learned' summary that will guide researchers and PAP leaders in increasing sustainability and fostering PAP leaders' use of research evidence – both in regard to how they can establish and nurture a strong and sustained working relationship, and in regard to how they can avoid potential pitfalls."

3. **Are the policy or practice issue(s) sensible ones to study? Is there a compelling rationale that research evidence plays any role (i.e., conceptual, instrumental, political, etc.) in those policy or practice issues?**

The area--PAPs that aim to improve behavioral health and/or promote well-being of youth aged 12-25 years--is one of the timeliest and costliest social problems in the U.S. There is a compelling rationale that evidence plays a role in this policy and practice issue. There is an equally strong rationale for research evidence to play a bigger role in the decision making of policymakers. Beyond this substantive policy issue, the issue of sustainable public-academic partnerships is timely and compelling and shows potential for leveraging improved outcomes.

4. **Does the applicant propose clear and compelling research questions and/or hypotheses?**

The overarching research questions clearly relate to use of research evidence: 1) How do PAPs develop and evolve over time in ways that support PAP leaders' use of research evidence? 2) What contexts and mechanisms of PAPs promote or inhibit PAP leaders' use of research evidence as PAPs evolve through life stages?

Additionally, exploratory questions 3 and 4 (below) and their sub-questions clearly relate to use of research evidence, including decision-making:

3) Are there different patterns of use of research evidence (in terms of obtaining, evaluating, and using research evidence) associated with each PAP life cycle?

4) Which PAP factors promote or inhibit PAP leaders' use of research evidence (in terms of obtainment, evaluation and actual use of research evidence)?

a) Does PAP leaders' use of research evidence differ by PAP partners' perceptions of alignment between PAP structure, goals and primary function, and process of setting agenda and their own organizational structure, goals and primary function, and process of setting agenda?

b) Do PAP partners' perceptions of interdependence, perceived mutual benefits, salience of the issue of focus, balanced power, top management support, convener's role, on-the-spot decision-making power, and partnership synergy have an impact on PAP leaders' use of research evidence

Given the state of science about public-academic partnerships, and the benefit they offer for increased use of evidence in youth serving systems, studying those factors that promote or inhibit public-academic partnerships' effectiveness would make a contribution to the field. The clarity of research questions is improved in this resubmission. They are clear and compelling.

RESEARCH DESIGN, METHODS, AND ANALYSES

1. Research Design and Methods

1a. Comment on the strength of the research design and sampling plan for addressing the research questions and/or hypotheses. Consider the sampling of cases, sites, individuals, documents, and so on.

The overall research design is better elaborated in this resubmission. I think the design is appropriate for addressing the research questions as they are articulated.

My only outstanding query is about the sampling of PAPs. Inclusion criteria are described as: "PAPs will be included if their aims include improving behavioral health and/or well-being outcomes for youth aged 12-25 years." Given (perhaps) the desire of this study to align with Interest c), above, which explicitly addresses "conditions under which using high-quality research improves decision making and youth outcomes."

1b. Comment on the data collection plan, including the strength of the data collection tools (i.e., interview or observation protocols, coding schemes, measures, etc.) and the plans to field those tools.

The data collection plan is well articulated in this resubmission. Survey tools are included and described sufficiently. Additional details were provided about CMO document analysis frameworks; the overall theory guiding case building; and the relationship between research questions and processes/products. Use of Palinkas' et al's Structured Interview for Evidence Use (SIEU) and Palinkas' inclusion as consultant is a strength of the proposal.

1c. If the applicant proposes measurement development work, comment on (i) the importance of the construct, (ii) the utility of the measure for the field, (iii) the rigor of the measurement work, and (iv) the team's measurement expertise.

No measurement development specified

1d. Comment on the feasibility of the methods and data collection plan. Many applicants propose to collect sensitive data from policymakers and practitioners. Are you persuaded that the team can successfully collect that data? For example, do they provide evidence that they have successfully done so in the past? Do they have convincing strategies for maximizing response rates and gaining access to respondents, meetings, and documents?

I am persuaded that the team can successfully collect the data specified in the proposal. Given that academic researchers and public administrators are fairly available, the team has convincing strategies for maximizing response rates and gaining access to respondents.

2. Data Analysis and Interpretation

2a. Comment on the sophistication and rigor of the analysis plan. Consider whether the team demonstrates an understanding of the strengths and limits of various analytic techniques.

This study is largely exploratory. Comparing cases across life cycle stages may also produce meaningful results. A plan is elaborated for coding, and I thought the analysis plan was much improved in this re-submission.

3. Do you have other comments on the significance or rigor of the proposed work?

This resubmission does a good job navigating the epistemological risks addressed in the last round of review by recasting the study questions to explore rather than impose the formation of PAP stage/life cycles.

ADDITIONAL CONSIDERATIONS

1. Is the budget appropriate? Does it reflect a cost-effective use of the Foundation's limited resources? Describe any concerns.

The budget is reasonable for each year. Personnel and knowledge translation costs are reasonable and expected.

2. Is the project team adequately staffed to successfully carry out the project? If not, what staffing or expertise is needed? (Please note that the Foundation discourages large advisory committees. Each proposed advisor should serve a specific and significant need for the project.)

The project team is strong and I believe adequately staffed to carry out the project. The PI clearly has ample experience as a researcher and ample preparation to conduct this study, despite no publications in this specific area. Additional detail was provided on the roles of PI and co-PI. Use of consultants is judicious and complements the project overall.

RESEARCH GRANTS EXTERNAL REVIEW

Review #3

**Foundation Focus Area**	Use of Research Evidence
**EGID**	188269
**Principal Investigator:**	Christina Kang-Yi
**Organization:**	University of Pennsylvania

THEORETICAL AND EMPIRICAL RATIONALE

1. Fit with the Foundation's Interests. Comment on the proposal's fit with the Foundation's interest in improving the use of research evidence. We support research to do any of the following: 1) identify, create, and test the structural and social conditions that foster more routine and constructive uses of existing research evidence; 2) identify, create, and test the incentives, structures, and relationships that facilitate the production of new research evidence that responds to decision makers' needs; and 3) investigate whether and under what conditions using high- quality research improves decision making and youth outcomes.

This proposal generally aligns with the Foundation's interest in improving the use of research evidence in that it seeks to understand development/evolution public-academic partnerships and the contexts within which PAP's influence/impact policy, practice, and outcomes. The work proposed offers systematic review of a sub set of partnerships through a conceptual framework that builds upon prior work in this arena. The results of this type of study have the potential to inform stakeholders about what works and what might not work when it comes to building and fostering partnerships and what supports application of research evidence by leaders and program directors in public agency.

2. Explain whether the applicant has chosen sensible policy or practice issues to study. Is there a compelling rationale that research evidence plays any roles on those issues?

There is a both strong belief and some evidence that research/practice partnerships and other types of partnerships between public agencies (in a variety of arenas) and researchers/scholars and academic centers matters and can have important impacts on policy and practice. So yes, it is well worth investing in understanding how these relationships work and if there are lessons learned for stakeholders (funders, scholars, leaders in public agencies) that can help strengthening or shape ongoing and future partnerships. It is also worth asking what the limits of the relationships are and should be.

3. Does the proposal reflect strong understanding of the would-be research users (i.e., policymakers, program managers, intermediaries, etc.) and of their work?

If not, describe how the understanding could be strengthened. Consider whether the applicant demonstrates a strong grasp of who makes key policy or practice decisions, how they make those decisions, and the ways organizational, political, or fiscal forces influence their work.

The applicant acknowledges the complexity that can be involved in applying evidence to practice and/or policy though, do not go into enough detail to allow reviewer to assess their understanding of the role and agency that would be research users or of their work. It will be important that the PI's on this effort, understand the local and regional contexts of the public agencies. Application of research evidence is no doubt challenging to understand...there is often research available to policy makers and program leads, and other stakeholders, but shifting toward employing that research evidence in a systematic way to improve outcomes is often a messy and complicated process that often requires agency leadership and political will as well as other architecture outside of a PAP to make it a success.

4. Does the applicant propose clear and compelling research questions and/or hypotheses?

The research questions seem to build upon those that came before and will make a contribution to the scholarship in this arena assuming the questions can be answered by the data they seek to collect. PI's outline clear table with where data will be collected to address which components of the research questions...

RESEARCH DESIGN, METHODS, AND ANALYSES

1. Comment on the feasibility of the methods and data collection plan. Many applicants propose to collect sensitive data from policymakers and practitioners. Are you persuaded that the team can successfully collect that data? For example, do they provide evidence that they have successfully done so in the past? Do they have convincing strategies for maximizing response rates and gaining access to respondents, meetings, and documents?

The proposal includes a list of 53 potentially eligible PAP’s constructed through preliminary literature reviews and searches (search criteria/methodology was not specified). Some PAP’s on the list appear to be training partnerships and/or direct care or treatment services that an academic center may be providing. These partnerships may not always have a traditional academic researcher as a lead. PAP's are defined in the proposal as having an academic researcher attached to them. Will the researchers include partnerships that are more akin to a traditional contract for a particular service or deliverable? Some additional clarity on sample selection criteria would be recommended prior to launching this project. Another area that may need some clarification is on which partnerships meet selection criteria. While the proposal indicates that as long as the aims include "improving behavioral health and/or well-being outcomes for youth aged 12-25," the PAP may be included. In some cases, these outcomes or aspirations may be rather distal to the intervention or partnership.

The applicant plans to do additional outreach to expand the potential eligible list, and will likely need to do so as their goal is to have 40 PAP's included in the study. The applicant identifies some reasonable strategies to broaden the sample, and indicated confidence that participation by PAP leaders would not pose a challenge. It is possible that the researchers are underestimating the challenge in achieving participation of PAP leaders given that they define the leaders as those who sit in the public agency. Depending on the public agency or State or jurisdiction, there may be some internal processes or procedures in place to gain

approval to participate in a study which could potentially delay engagement by PAP leaders. PAP leadership may be more subject to turnover or internal movement than academic researcher turnover and it is not clear what strategies the researchers would employ, if any, to locate a former PAP leader or what strategies they will use to engage an agency to secure their participation on the research project. It is also often the case that public agencies are not always interested or well positioned to share non-public documents with researchers they do not know. While there is a wealth of experience on the research team associated with this proposal, there is little to no acknowledgment of some of the potential barriers they may face gaining access and participation of the public agency partner/PAP leader or strategies to overcome them.

Additionally, the project focuses on PAP's that were formed to improve behavioral health and/or promote well-being of youth age 12-25; PAP's focused on this subset of youth/young adults are likely served by both adult and child/youth serving systems. And while this reviewer understands this age range is very much within the priority of the Foundation, the applicant doesn't provide clear rationale for focus on this particular age range which may complicate PAP selection process.

2. Do you have other comments on the significance or rigor of the proposed work?

It is not altogether clear how this proposal would build upon knowledge or strategies for reducing inequality in youth outcomes given the research design, also a major goal of the Foundation though not one necessarily required to be of focus for this funding opportunity. For example, there is no indication in the proposal that the partnerships will be analyzed for their ability or demonstrated capacity to reduce inequality in outcomes and/or improve outcomes.
